# Extraction, purification, structural features, biological activities, and applications of polysaccharides from *Opuntia ficus-indica (L.)* Mill. (cactus): a review

**DOI:** 10.3389/fphar.2025.1566000

**Published:** 2025-03-12

**Authors:** Xudong Liu, Yan Xing, Guijun Liu, Dapeng Bao, Wenjing Hu, Haizheng Bi, Meng Wang

**Affiliations:** ^1^ Key Laboratory of Basic and Application Research of Beiyao Ministry of Education, Heilongjiang University of Chinese Medicine, Harbin, China; ^2^ Nursing Humanities Teaching and Research Office, Heilongjiang Nursing College, Harbin, China

**Keywords:** Opuntia ficus-indica (L.) Mill., polysaccharides, structural features, biological activities, applications

## Abstract

Cactus has attracted increasing attention from researchers due to its rich nutritional, edible, and medicinal value. Cactus contains abundant polysaccharides, polyphenols, vitamins, amino acids, minerals, and more. Among them, polysaccharides are considered as important bioactive components in cactus. In the past period, polysaccharides have been isolated from cactus through various methods and their structures have also been studied. Some *in vivo* and *in vitro* experimental results indicate that cactus polysaccharides have promoting wound healing, anti-inflammatory, immune regulation, anti-glycosylation, and antioxidant effects. This article reviews the research progress in the extraction, purification, structural characteristics, and biological activities of cactus polysaccharides in recent years. In addition, the relationship between the structure and activity of cactus polysaccharides was also discussed. This review provides important research basis and latest information for the in-depth development and application of cactus polysaccharides in multiple fields such as medicine and functional foods.

## Graphical Abstract

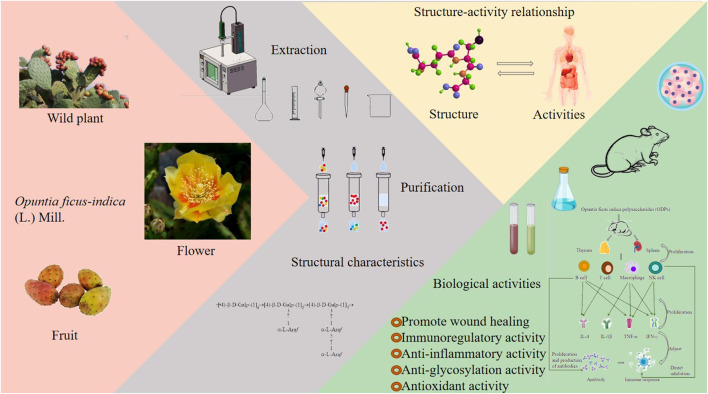

## 1 Introduction

**FIGURE 1:**
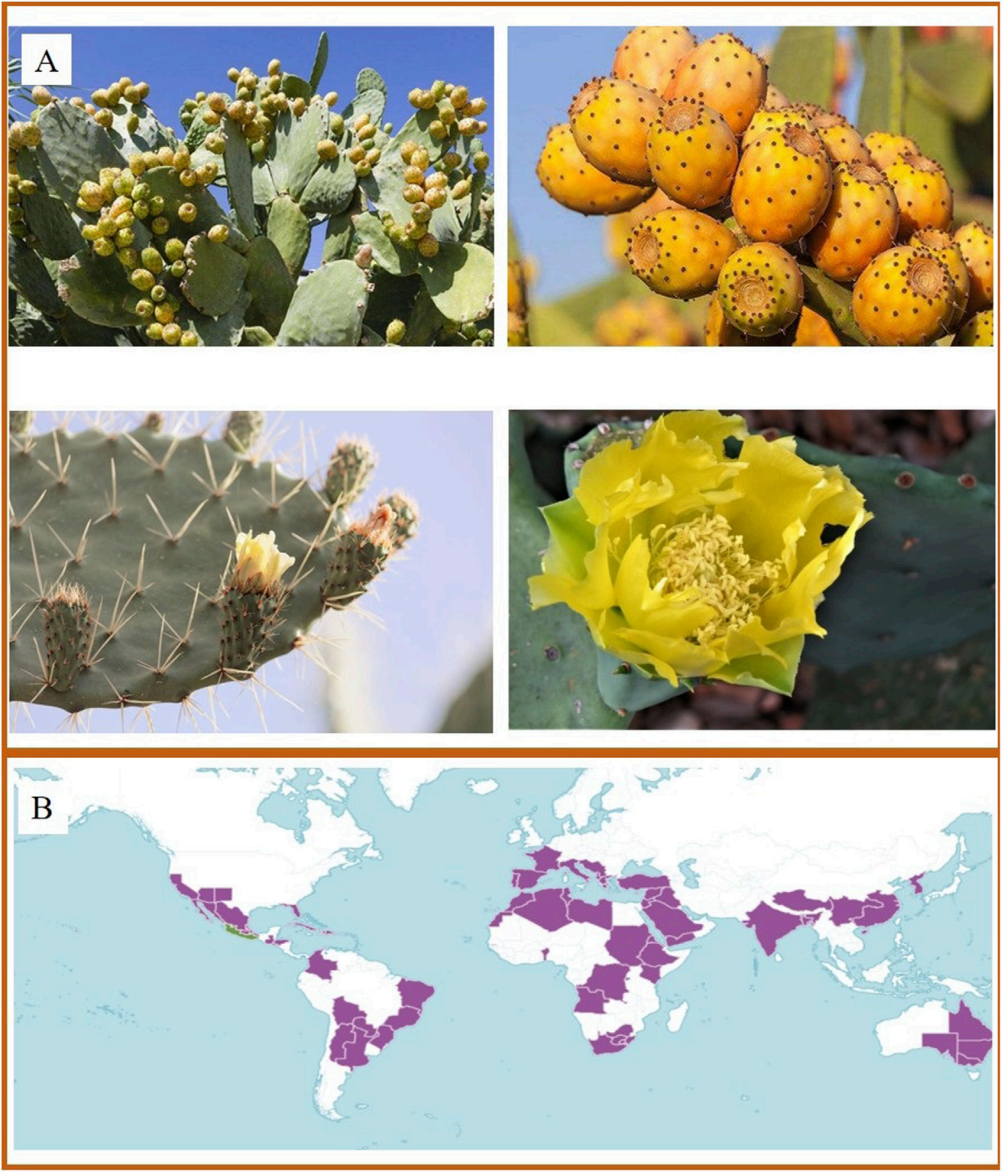
**(A)** The morphological characteristics of cactus. (For intuitive introduction to the plant morphology of cactus, the images are processed and quoted to show the characteristics of different parts from network and public sources). **(B)** World distribution of cactus. (https://powo.science.kew.org/taxon/urn:lsid:ipni.org:names:1151735-2).

*Opuntia ficus-indica* (L.) Mill. (cactus) is a fleshy shrub of the genus *Cactus* in the family Cactaceae. It is 1.5–5 m tall and has several wide broad oval branches of light green or gray green in colour, and its flowers are orange red. Its fruit looks like a pear, so it is also called pear cactus (Figure 1). Its well-developed root system and fleshy stems make it extremely viable, able to thrive in environments with high temperatures, high carbon dioxide levels, and low rainfall (Reyes-Agüero et al., 2005; Wang et al., 2023). Moreover, growing cactus in large quantities can greatly reduce the evaporation of soil moisture, which is beneficial for windbreak and sand fixation (Naorem et al., 2024). Cactus is native to Mexico and has been a domesticated plant in Latin America, Africa, Mediterranean countries, and the Middle East. Under the current situation of global climate change and the increasing environmental crisis, the strong environmental adaptability and diversity value of cactus deserve more attention.

Cactus is a versatile plant. Each part of cactus has extremely high nutritional value. According to the research, every 100 mg cactus cladode contains 220–320 mg of calcium, 52–85 mg of magnesium, 300–430 mg of potassium, 21–45 mg of sodium, and 0.5–2.0 mg of iron. The amount of the above minerals per 100 mg cactus fruit is less than that of cladode (Monteiro et al., 2023). In addition, cactus has the ability to survive in extremely harsh desert environments, which is attributed to its strong water holding capacity. It is reported that the water content of fresh cactus cladode accounts for about 90%–94% of its total fresh weight. At the same time, the cladode of cactus is also rich in dietary fiber, vitamins C and E, and is regarded as an ideal healthy food (Peña-Vázquez et al., 2025). In Mexico, the young cladodes of cactus, also known as nopalitos, are consumed as vegetables for cooking or roasting. Cactus can also be consumed as fruit juice, jam, jelly, wine, vinegar, and other processed products. These products are widely used in Latin America. Moreover, cactus contains 8 kinds of essential amino acids required by the human body, and the proportion of essential amino acids is appropriate, which meets the human body’s needs for human food nutrition. Besides essential amino acids, cactus also contains 17 other amino acids (Table 1) (El-Mostafa et al., 2014). In addition to its many nutritional benefits, cactus has surprising medicinal properties. The fruit and cladodes of cactus are traditionally used to treat diabetes, hypertension, burns, edema, and indigestion in oriental folk medicines (El-Hawary et al., 2020). The fruit of cactus can be used as a laxative in Turkey and can also be used to relieve the pain of rheumatism and kidney stones (Gürdal and Kültür, 2013; Akkol et al., 2020). In addition, in Chinese folk, cactus is used to accelerate the healing of wounds and ulcers and treat gastrointestinal diseases and atherosclerosis (Giraldo-Silva et al., 2023).

**TABLE 1:** Distribution and contents of amino acids in cladode, fruit, and seeds from cactus.

Amino acid	Cladode (g/100 g)	Fruit (g/100 g)	Seeds (g/100 g)
Alanine	1.25	3.17	4.75
Arginine	5.01	1.11	6.63
Asparagine	3.13	1.51	NA
Asparaginic acid	4.38	NA	10.42
Glutamic acid	5.43	2.40	21.68
Glutamine	36.12	12.59	N/A
Cystine	1.04	0.41	0.37
Histidine	4.18	1.64	3.11
Isoleucine	3.97	1.13	6.20
Leucine	2.71	0.75	9.94
Lysine	5.22	0.63	6.79
Methionine	2.92	2.01	0.70
Phenylalanine	3.55	0.85	5.25
Serine	6.68	6.34	8.46
Threonine	4.18	0.48	1.53
Tyrosine	1.46	0.45	3.09
Tryptophane	1.04	0.46	NA
Valine	7.72	1.43	6.02
*α*-Aminobutyric acid	NA	0.04	NA
Carnosine	NA	0.21	NA
Citrulline	NA	0.59	NA
Ornithine	NA	NA	NA
Proline	NA	46.00	NA
Taurine	NA	15.79	NA
Glycine	NA	NA	5.06

Abbreviations: NA = not available.

Note: Data adapted from El-Mostafa et al., 2014.

In recent years, a large number of research reports on cactus have shown that they have hypoglycemic, antibacterial, and neuroprotective effects, which are attributed to the various bioactive substances contained in cactus (Bensadón et al., 2010; Missaoui et al., 2020). Traditionally, small molecule compounds in cactus are considered their main active ingredients. However, with the improvement of scientific research level, it has been discovered that the macromolecular compound cactus polysaccharide is also an important active substance (Palmieri et al., 2021; Peng et al., 2023). The polysaccharides obtained from cactus have surprising and ideal biological activities, including promoting wound healing, immune regulation, anti-inflammatory, anti-glycosylation, and antioxidant activities (Deters et al., 2012; Lefsih et al., 2016; Liu et al., 2022; Adjafre et al., 2024). By using different extraction methods to separate different cactus polysaccharides, they have diverse structures and rich biological activities, providing more possibilities for the development of new drugs or functional foods. Meanwhile, cactus polysaccharides have excellent antioxidant and moisturizing effects and can also be used as raw materials for cosmetics and toiletries.

With people’s attention to diet health and physical health, cactus as a special food has been widely concerned. It has not only edible value and nutritional value but also medicinal value. Polysaccharides obtained from cactus just meet the needs of the body for health functions. Referring to the existing literature, there are some articles on cactus polysaccharides, mainly focusing on the extraction and purification of polysaccharides and pharmacological effects, lack of a comprehensive review of cactus polysaccharides. In this review, the research progress on extraction and purification methods, structural characteristics, health benefits and product applications of cactus polysaccharides were reviewed. In addition, the structure-activity relationship of cactus polysaccharides was also emphasized in order to provide valuable insights for further study of cactus polysaccharides.

## 2 Extraction and purification of cactus polysaccharides

There are various methods for extracting natural polysaccharides. Solvent extraction is a traditional method, including water extraction, hot water extraction (HWE), acid extraction and alkaline extraction. At present, many novels environmentally friendly technologies, such as ultrasound-assisted extraction (UAE), microwave-assisted extraction (MAE), enzyme-assisted extraction (EAE) and calcium chelator extraction, have been reported for the effective and selective extraction of cactus polysaccharides.

### 2.1 Extraction method of cactus polysaccharides

#### 2.1.1 Traditional extraction method

Solvent extraction is the most used method to extract polysaccharides from plants. Deters et al. (2012) obtained polysaccharides (NwPS) from cactus at 4°C with water as extraction solvent, and the extraction rate was 2.74%. HWE is a traditional method that has been recognized and widely used due to its ease of operation and economic advantages (Duan et al., 2018). Lefsih et al., (2015) used HWE method to extract polysaccharides (WSP-d) from cactus, and the extraction rate was 5.75%. Although HWE has low cost and minimal equipment requirements, it has disadvantages such as long processing time, low extraction efficiency, high extraction temperature, and possible degradation of polysaccharides (Wang et al., 2019). Various methods have been developed and improved to improve the yield of polysaccharides. Cactus polysaccharides PC was extracted by adding hydrochloric acid to adjust the pH of the solution to 2.8, heating it in a water bath at 90°C for 2 h, and the yield was about 6.13% (Bayar et al., 2016). In addition to using acid reagents for extraction, alkaline reagents can also be used. When extracting with alkaline reagents, ammonium oxalate and sodium hydroxide are often preferred. It is worth noting that the acid and alkali solvent extraction methods have limitations; during the extraction process, the spatial structure of cactus polysaccharides is easily destroyed under acidic conditions, and some polysaccharides are easily degraded under strong alkaline conditions (Habibi et al., 2002). Therefore, the selection of suitable conditions has an important influence on polysaccharides extractions.

#### 2.1.2 Novel extraction method

With the deepening of research, some novel extraction techniques have been gradually developed to obtain polysaccharides. UAE is an efficient and practical polysaccharides extraction method, which mainly uses the mechanical effect of ultrasound to destroy the cell wall and increase the mass transfer efficiency in cells, thereby promoting the release and extraction of plant polysaccharides. However, the extraction efficiency of UAE is also affected by many factors, including but not limited to time, temperature, and solid-liquid ratio (Xue et al., 2024). Studies have reported that the yield of cactus polysaccharides obtained by UAE varied between 2.45% and 5.24% when the extraction temperature was increased from 30°C to 70°C (Bayar et al., 2017). Compared with the traditional extraction methods, it has the advantages of short extraction time and low energy consumption. MAE uses microwave radiation to obtain a large amount of heat from the polar substances in plant cells, causing the intracellular temperature to rise. The pressure generated by the vaporization of liquid water leads to the rupture of the cell membrane and cell wall, forming tiny holes, and thus the plant polysaccharide components in the cells are released. It has the advantages of fast heating, strong penetration and short extraction time (Niu et al., 2024). Lefsih et al., (2016) used MAE to extract polysaccharides WSP-f from cactus, and the extraction conditions were optimized by response surface methodology. The optimal conditions were as follows: extraction time was 2.15 min, microwave power was 517 W, pH was 2.26, solid-liquid ratio was 2g/30.6 mL, and the extraction yield was 12.57% (Lefsih et al., 2017). Enzymes such as xylanase enzyme and cellulase enzyme can accelerate the release and extraction of polysaccharides from cactus samples by removing mucus. EAE method has the characteristics of mild conditions, easy removal of impurities and high recovery rate, which has broad development prospects (Zhang et al., 2024). Bayar et al., (2018) obtained polysaccharides EAEPC with a yield of 17.91% at a liquid-to-solid ratio of 22 mL/g, a ratio of cellulase to xylanase of 2 : 1, and an enzymes-matter ratio of 4 U/g (Bayar et al., 2018). Ethylene Diamine Tetraacetic Acid (EDTA) disodium is the most commonly used reagent in calcium chelator extraction. Habibi et al. (2004a) obtained polysaccharide CSP from cactus by sequential extraction with EDTA solution at 60°C. In addition, if a single extraction method cannot meet the needs, two or more composite methods can also be selected, such as ultrasonic microwave-assisted extraction, ultrasonic enzyme-assisted extraction and microwave enzyme-assisted extraction.

### 2.2 Purification method of cactus polysaccharides

Polysaccharides obtained from natural plants often have impurities such as proteins, pigments, and small molecular substances, which can affect the structural features and biological activities of the polysaccharides. Sevag method is a commonly used method for protein removal; the principle is that n-butanol and chloroform precipitate proteins (Ke et al., 2023). Pigments can be removed from polysaccharides by chemical and physical methods such as peroxidation and reduction. Their mechanism of occurrence is that the structure of the polysaccharide molecule is changed through chemical reactions, so that its color disappears (Rao et al., 2024). Although the physical method does not require the use of chemical reagents and has little effect on the structure and properties of polysaccharides, the decolorization effect may not be good enough. Small molecule compounds were removed by dialysis and then crude polysaccharides were obtained. Furthermore, further purification of polysaccharides can be obtained by hierarchical precipitation, gel chromatography, cellulose column chromatography, ion-exchange cellulose chromatography and ultrafiltration. Their principle is polarity difference, adsorption and molecular sieving effect with low energy consumption, easy recovery and reuse of the eluent and the advantages of high purity of the resulting product. Finally, the purified polysaccharides were obtained by concentration and drying. The extraction methods, time, temperature, solid-liquid ratios, total rates, purification methods and other information of cactus polysaccharides are summarized in Table 2.

**TABLE 2:** A summary of cactus polysaccharides extraction and purification methods.

Polysaccharide fraction	Extraction					Purification		Ref
	Extraction methods	Time (h/min)	Conditions	Solid–liquid ratio (g/mL)	Total yield (%)	Polysaccharide fraction	Purification methods	
CP	Water extraction	2 h	Room temperature	NA	4.1%	FI-FV	DEAE-Sepharose, and CL-6B column	Habibi et al. (2004b)
CSP	Calcium chelator extraction	2 h	60°C	NA	12.4%	CSP1-5	EtOH precipitation, and DEAE Trisacryl-M	Habibi et al. (2004a)
CSP-d	Calcium chelator extraction	1.5 h	80°C	1 : 20	0.21%	ASP	NaOH neutralization, and EtOH precipitation	Lefsih et al. (2016)
Crude polysaccharide (dried fruits)	Hot water extraction	6 h	100°C	NA	0.65%	PS-1	DEAE-Sephadex A-25 column, and Sephadex G-200 column	Ishurd et al. (2010)
d-xylans	Alkaline extraction	4 h	100°C	NA	NA	F1∼F6	EtOH precipitation, and polyacrylamide Biogel P6 column	Habibi et al. (2002)
EAEPC	Enzyme-assisted extraction	NA	NA	1 : 22	17.91%	NA	Isopropanol precipitation, and freeze drying	Bayar et al. (2018)
NwPS	Water extraction	NA	4°C	1 : 5.81	2.74%	NA	EtOH precipitation, centrifugation, and dialysis	Deters et al. (2012)
ODPs	Hot water extraction	3 h	90°C	1 : 50	9.20%	NA	EtOH precipitation, centrifugation, and freeze drying	Liu et al. (2022)
(OFIFG)s	Microwave-assisted extraction	NA	NA	1 : 10	NA	NA	Filtered and freeze-drying	Salehi et al. (2019)
PC	Acid water	2 h	90°C	1 : 15	6.13%	NA	NA	Bayar et al. (2016)
PCA	Ultrasound-assisted extraction	10 min	NA	NA	1.3%	NA	EtOH precipitation, freeze drying, and dialysis	Chaouch et al. (2016)
PCB	Ultrasound-assisted extraction	10 min	NA	NA	0.7%	NA	NA	Chaouch et al. (2016)
PCN	Ultrasound-assisted extraction	10 min	NA	NA	3.5%	NA	NA	Chaouch et al. (2016)
TPL-Ofi	Alkaline extraction	6 h	90°C	NA	0.33%	NA	EtOH precipitation, dialysis, and centrifugation	Adjafre et al. (2024)
UAEPC	Ultrasound-assisted extraction	70 min	70°C	1 : 30	18.14% ± 1.41%	NA	EtOH precipitation	Bayar et al. (2017)
WSP	Hot water extraction	2 h	60°C	NA	6.1%	WSP1-5	EtOH precipitation, and DEAE Trisacryl-M	Habibi et al. (2004a)
WSP-d	Hot water extraction	1.5 h	60°C	1 : 10	5.75%	NA	EtOH precipitation	Lefsih et al. (2016)
WSP-f	Microwave-assisted extraction	2.15min	NA	1 : 15.3	12.57%	NA	Oven drying	Lefsih et al. (2017)
WSP-k	Microwave-assisted extraction	2.16 min	517 W	1 : 14.3	NA	dWSP, HM-dWSP	EtOH precipitation, freeze drying, and deproteinization	Lefsih et al. (2018)

Abbreviations: NA = not available.

## 3 Chemistry of cactus polysaccharides

**FIGURE 2:**
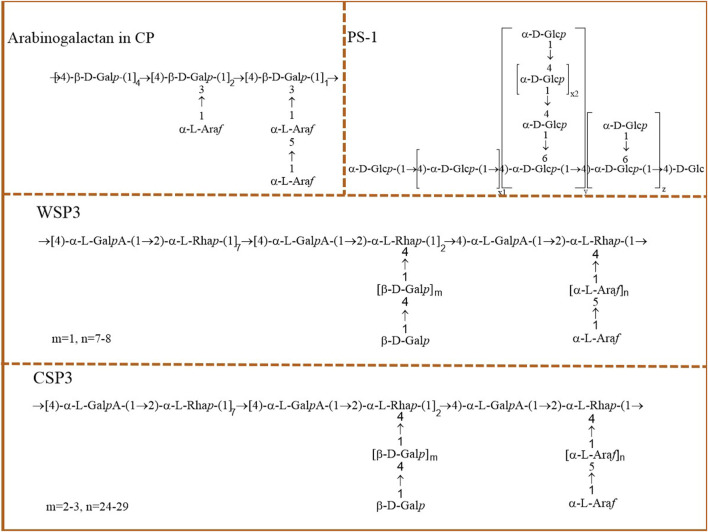
The hypothetical structure of cactus polysaccharides.

Different extraction and purification methods can lead to differences in the chemical structures and physicochemical properties of polysaccharides. The biological activity of plant polysaccharides is closely related to their molecular weight, monosaccharide compositions, the way of glycosidic bond connection, and the basic configuration of the main side chain (Chen et al., 2020; Geng et al., 2024). Therefore, it is important to explore the structure of plant polysaccharides for studying their biological activities and potential structure-activity relationships. According to previous literature, a variety of cactus polysaccharides have been gradually found to have different structures and properties. We present comprehensive information on cactus polysaccharides in Table 3 regarding the name, molecular weight, monosaccharide compositions, structure, and relevant references of each compound. The reported polysaccharide chemical structures are shown in Figure 2.

**TABLE 3:** Source, compound name, molecular weights, monosaccharide composition, structures of cactus polysaccharides, and analytical techniques.

Source	Compound name	Molecular weights	Monosaccharide composition	Structures	Analytical techniques	Ref
Cladodes	ASP	NA	Gal: Glc: GalA: Ara: Rha: Man = 40.2 : 7.3 : 44.3 : 0.8 : 6.6 : 0.8	ASP fraction, suggesting that the chains of galactan are related to the insoluble parietal polysaccharides	FT-IR, HPAEC, GC-MS	Lefsih et al. (2016)
	CSP		Gal: Glc: GalA: Ara: Xyl: Man = 8.2 : 1.9 : 81.1 : 4.1 : 0.5 : 4.0	NA	NA	Lefsih et al. (2016)
	dWSP		NA	dWSP is constituted of the main polyrhamnogalacturonate backbone with different side chains made up of neutral sugars, mainly glucan and galactan	GC, HPSEC	Lefsih et al. (2018)
	EAEPC	60.42 kDa		This result involved the presence of the rhamnogalacturonan I domain in the EAEPC structure. EAEPC is low methylated pectin	TLC, IR	Bayar et al. (2018)
	PC	110.70 ± 3.37, 78.40 ± 5.49, 58.57 ± 4.31 kDa		PC has free carboxyl groups and *α*-glycosidic linkages	FT-IR, HPLC	Bayar et al. (2016)
	TPL-Ofi	227.781 kDa		The FT-IR analysis showed that the TPL-Ofi contained methyl and carboxyl groups	NA	Adjafre et al. (2024)
	WSP-d	NA	Gal: Glc: GalA: Ara: Rha: Man = 11.4 : 6.9 : 66.6 : 9.2 : 2.1 : 3.8	NA	GC-MS, FT-IR	Lefsih et al. (2016)
Fruits	OFIFG	3.67 × 10^3^ kDa	Glc: Ara: Xyl: Gal: Man = 78.0 : 12.9 : 4.8 : 2.4 : 2.4	COOCH_3_ group in OFIFG structure	FT-IR, NMR, GC-MS, ICP-OES	Salehi et al. (2019)
	PS-1	∼360 kDa	NA	Polysaccharide PS-1 was found to be an *α*-d-glucan with a (1→4)-linked *α*-d-Glc*p* backbone, with (1→6)-linked (1→4)- *α*-d-Glc*p* side chains, side chains being short in length with no additional branching, and a minimum branching of ∼1 in every 9–11 backbone units	GC–MS, Methylation analysis	Ishurd et al. (2010)
Fruit peel	CP	NA	GalA: Rha: Glc: Gal: Ara: Xyl: Man = 14.2 : 4.2 : 1.5 : 23.5 : 32.7 : 4.5	NA	NMR, SEM, HPSEC	Habibi et al. (2004a)
	CSP		Rha: Gal: Ara: Xyl = 3.7 : 4.0 : 17.2 : 0.9		NA	Habibi et al. (2004a)
	CSP1		Glc: Gal: Ara: Xyl = 4.1 : 50.1 : 43: 2.3			Habibi et al. (2004a)
	CSP2		GalA: Rha: Glc: Gal: Ara: Xyl = 18 : 5.2 : 1.4 : 11.4 : 50.2 : 1.1			Habibi et al. (2004a)
	CSP3		GalA: Rha: Glc: Gal: Ara: Xyl: Man = 29.4 : 7: 2:12 : 37: 2.5 : 1	CSP3 consisted of repeating units →2)-*α*-l-Rhap-(1→4)-*α*-d-Gal*p*A-(1→, with 71% of rhamnose units linked in O-2 and 29% both in O-2 and O-4, and with side chains of either arabinan (average of 25–30 arabinose units) or galacto-oligosaccharides (average of 3–4 galactose units) attached to O-4 of the backbone rhamnose units		Habibi et al. (2004a)
	CSP4		GalA: Rha: Gal: Ara = 48.5 : 6: 5.2 : 10.3	NA		Habibi et al. (2004a)
	CSP5		GalA: Rha: Gal: Ara = 60.5 : 5: 2.5 : 7.5			Habibi et al. (2004a)
	FI	85 kDa	Rha: Glc: Gal: Ara: Xyl: Man = 1.1 : 1.9 : 65.5 : 30.1 : 1.3	FI is an arabinogalactan mainly composed of (1→4) -linked *β*-d-galactose residues, with 60.5% of free galactose units and 39.5% of galactose units being branched at position 3	NA	Habibi et al. (2004b)
Leaf	NwPS	NA	Ara: Gal: GalA: Glc: GlcA: Rha: Xyl = 15 : 20: 27 : 18: 2 : 11	NA	TLC, HPAEC	Deters et al. (2012)
	NPec		Ara: Gal: GalA: Glc: GlcA: Rha: Xyl = 9 : 14: 44 : 15: 8 : 1: 9		NA	Deters et al. (2012)
Pericarp seeds	F2	NA	Ara: Xyl: Glc = 2.2 : 80.7 : 3.9	F2 was assumed to be (4-O-methyl-d-glucurono)-d-xylans, with 4-O-d-glucopyranosyluronic acid groups linked at C-2 of a (1→4)-*β*-d-xylan	NMR	Habibi et al. (2002)
	F3		Xyl: Glc = 90.3 : 1.1	NA	NA	Habibi et al. (2002)
	F4		Xyl: Glc = 87.5 : 2.3	F4 is mainly composed of (1→4)-*β*-d-Xyl*p*, (1→4)-*β*-d-Xyl*p*-2-O-Glc*p*A and 4-O-Me-*α*-d-Glc*p*A residues		Habibi et al. (2002)

Abbreviations: glucose = Glc, galactose = Gal, rhamnose = Rha, arabinose = Ara, xylose = Xyl, mannose = Man, glucuronic acid = GlcA, galacturonic acid = GalA, not available = NA.

### 3.1 Molecular weights

The molecular weight (Mw) of bioactive polysaccharides is an important factor affecting their biological activity. At present, the methods for determining the Mw of polysaccharides mainly include high-performance liquid chromatography (HPLC), high-performance gel permeation chromatography (HPGPC), high-performance gel filtration chromatography (HPGFC) and gel filtration high performance liquid chromatography and ultrafiltration interception. The chromatographic method is simple, accurate and suitable for the determination of cactus polysaccharides. The Mw of polysaccharides from different parts is different. The Mws of FI and EAEPC isolated from cactus fruit peel and cladodes were 85 kDa and 60.42 kDa, respectively. Moreover, the average Mw of polysaccharides obtained from cactus was different by different extraction methods. The Mw of polysaccharides OFIFG obtained from cactus by MAE method was 3.67 × 10^3^ kDa, while the Mw of polysaccharides PS-1 extracted from cactus by HWE was ∼360 kDa (Salehi et al., 2019; Ishurd et al., 2010; Liu et al., 2022). Summarizing the existing literature, the average molecular weight of cactus polysaccharides was roughly in the range of 58.6–3.67 × 10^3^ kDa (Bayar et al., 2016; Salehi et al., 2019). In conclusion, in the determination of the average molecular weight of cactus polysaccharides, the difference in Mw may be related to the different extraction methods, separation and purification methods, and analytical methods of cactus.

### 3.2 Monosaccharide compositions

The monosaccharide compositions of cactus polysaccharides are also closely related to their biological activities. GC-MS and HPLC are commonly used to determine the composition of monosaccharides. The different raw materials, extraction and purification methods also had a remarkable effect on the monosaccharide composition of cactus polysaccharides. A summary of previous literature showed that polysaccharides obtained from cladodes, fruits, fruit peel, pericarp seeds, and leaves had different monosaccharide compositions, which were mainly composed of glucose (Glc), galactose (Gal), rhamnose (Rha), arabinose (Ara), xylose (Xyl), mannose (Man), glucuronic acid (GlcA), and galacturonic acid (GalA). Notably, polysaccharides extracted from cactus fruits and cladodes did not have GlcA in their monosaccharide composition, while polysaccharides isolated from leaves did not have Man (Habibi et al., 2004b; Deters et al., 2012; Lefsih et al., 2016). Monosaccharide composition analysis indicate that polysaccharides from cactus fruit peel (CP) is mainly composed of GalA, Rha, Glc, Gal, Ara, Xyl and Man, and the mole ratio is 14.2 : 4.2: 1.5 : 23.5: 32.7 : 4.5 (Habibi et al., 2004a). Interestingly, the polysaccharides isolated from the pericarp seeds were mainly composed of Ara, Xyl and Glc (Habibi et al., 2002). Furthermore, the monosaccharide composition of CP and CSP3 was similar, but the molar ratio was different. CP and CSP3 are polysaccharides from fruit peel and fruit, respectively. Both of them are mainly composed of GalA, Rha, Glc, Gal, Ara, Xyl and Man, and their molar ratios are 14.2: 4.2: 1.5: 23.5: 32.7: 4.5 and 29.4: 7: 2: 12: 37: 2.5: 1 (Habibi et al., 2004a; Habibi et al., 2004b). The monosaccharide composition of polysaccharides extracted from the same part is also very different. Habibi et al. (2004b) used HWE for repeated extraction at 60°C for 2 h to obtain crude cactus polysaccharides WSP, which was mainly composed of Rha, Glc, Gal, Ara, Xyl and Man. Subsequently, WSP was partially esterified and purified by anion exchange chromatography to give different fractions WSP2 and WSP3. In the analysis of monosaccharide composition, WSP2 has more GalA and less Man on the basis of WSP, while WSP3 increased GalA and lacks Glc, Xyl and Man (Habibi et al., 2004a).

### 3.3 Structural characteristics

The biological significance of polysaccharides is closely related to its complex structural characteristics and novel skeleton characteristics. Therefore, the study of chemical structure of polysaccharides is of great significance for the value-added utilization of cactus resources (Ji et al., 2023). The structural characteristics of cactus polysaccharides, such as glycosyl linkage type and branched chain type, were preliminarily studied and established by various chromatographic techniques and spectral analysis methods. FI is an arabinogalactan isolated from cactus crude polysaccharides CP by anion-exchange chromatography. Through methylation derivation and NMR analysis, the backbone of FI was found to be composed of (1→4) -linked *β*-d-galactose residues, and 39.5% of the galactose units were substituted at the O-3 position. These branches contain L-arabinose units and may also contain L-arabinose *α*-l-(1→5) l-arabinose linked disaccharides (Habibi et al., 2004b). A pectin polysaccharide WSP, was isolated by Habibi et al. from the fruit peel of cactus and purified by size exclusion chromatography to obtain WSP3. Structural analysis showed that WSP3 consisted of repeating units →2)-*α*-l-Rhap-(1→4)-*α*-d-Gal*p*A-(1→, with 71% of rhamnose units linked in O-2 and 29% both in O-2 and O-4, and with side chains of either arabinan or galacto-oligosaccharides attached to O-4 of the backbone rhamnose units (Habibi et al., 2004a). Polysaccharide dWSP obtained from cactus cladodes was constituted of the main polyrhamnogalacturonate backbone with different side chains made up of neutral sugars, mainly glucan and galactan (Lefsih et al., 2018). Cactus pericarp seed polysaccharides F4 is mainly composed of (1→4)-*β*-d-Xyl*p*, (1→4)-*β*-d-Xyl*p*-2-O-Glc*p*A and 4-O-Me-*α*-d-Glc*p*A residues (Habibi et al., 2002). Cactus fruit polysaccharides PS-1 is an *α*-d-glucan with a (1→4)-linked *α*-d-Glc*p* backbone, with (1→6)-linked (1→4)-*α*-d-Glc*p* side chains (Ishurd et al., 2010). The above summary and analysis of the structural characteristics of cactus polysaccharides provide valuable information for the structural study of cactus polysaccharides.

## 4 Biological activities of cactus polysaccharides

**FIGURE 3:**
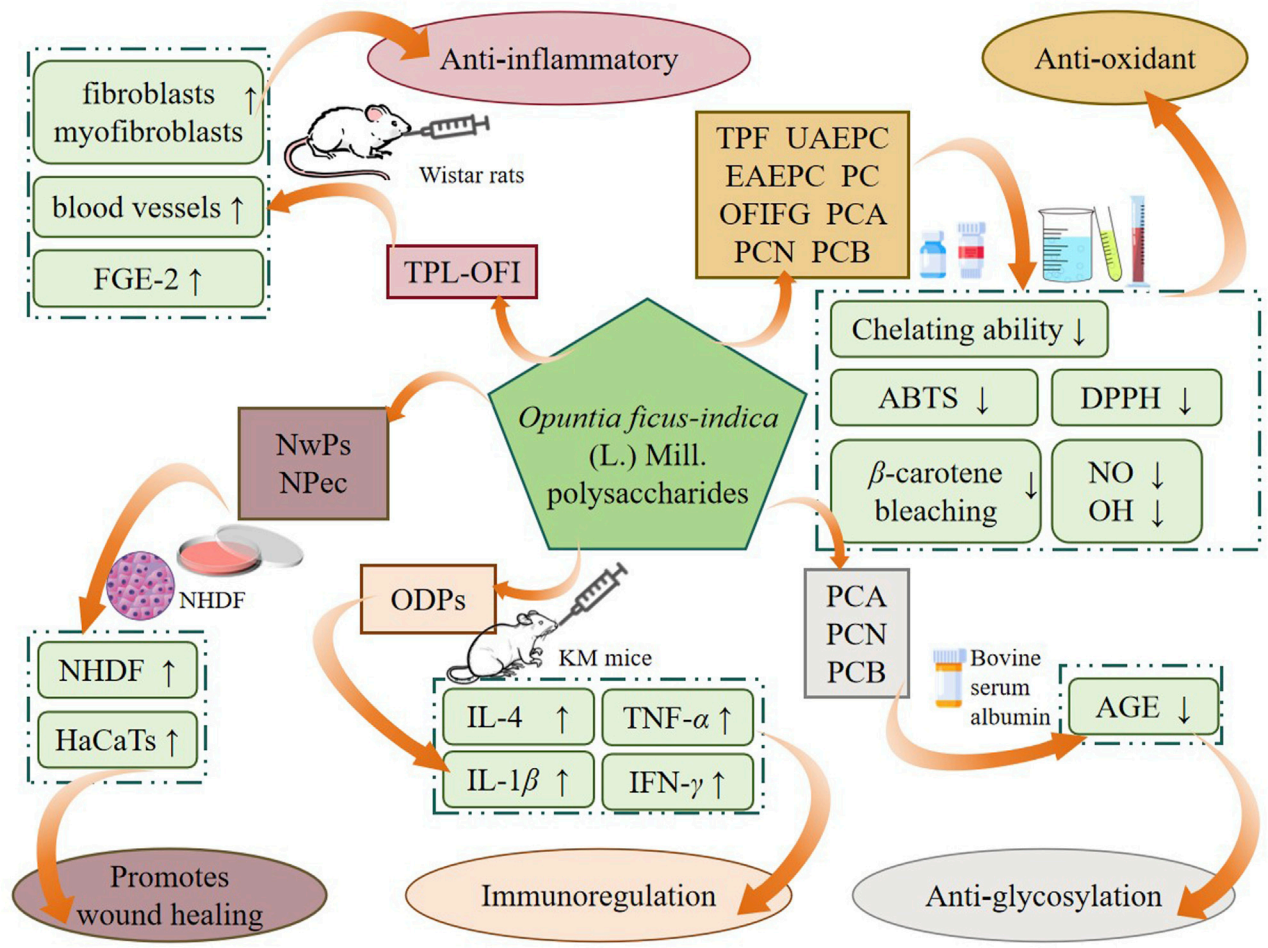
Biological activities of polysaccharides from cactus.

As an important nutrient and food source, cactus has high research and development value. As one of the active ingredients, cactus polysaccharides have biological activities such as promoting wound healing, anti-inflammatory, anti-glycation, immunoregulation and other activities. A number of *in vitro* and *in vivo* studies have been conducted to investigate the biological activities of cactus polysaccharides (Figure 3).

### 4.1 Promote wound healing

The incidence of skin wound is high, and the repair process is complex, which is easy to cause problems such as poor healing, resulting in a huge economic and social burden, and seriously affecting the quality of life of patients. Skin wound is one of the reasons that threaten human physical and mental health, and how to promote wound healing has always been the focus of attention and research (Byun et al., 2024; Rathna and Kulandhaivel, 2024). Keratinocyte (KC) are the main components of epidermis, while fibroblasts are the main components of dermis. They are closely related to wound healing. Research has shown that cactus polysaccharides have a positive effect on wound healing and regeneration. However, the effects of water-soluble cactus pear polysaccharide NwPS and no expansive pectin NPec on skin fibroblasts and keratinocyte are different due to the difference in the contents of glucose and Uronic acid. At 0.1 μg/mL, 1 μg/mL, and 100 μg/mL, NwPS significantly stimulated the metabolic activity of HaCaT. When the concentration of NwPS was 0.1 μg/mL and 10 μg/mL, the metabolic activity of HaCaTs incubated with NwPS was significantly different with the different incubation time. However, NPec had no significant effect on the metabolic activity of HaCaTs after incubation for 48 and 72 h. Significant effects were only observed when HaCaTs were incubated with 100 μg/mL NPec for 48 h. KC were incubated with 1 mg/mL and 10 mg/mL NPec respectively, and the proliferation rate was observed. Compared with the proliferation rate measured after 48 h of incubation, the proliferation rate increased significantly but not significantly after 72 h (Deters et al., 2012). These basic studies have laid a solid foundation for the further development and utilization of cactus polysaccharides in the fields of dermatology and cosmetics.

### 4.2 Anti-inflammatory activity

When the skin is injured, if not properly handled, it is easy to induce infection and cause local inflammation (Farasati Far et al., 2024). Cactus polysaccharides TPL-ofi was isolated and extracted with alkaline water, and its good anti-inflammatory effect was verified. A biopsy punch (8 mm in diameter) was used to create four circular, full-thickness wounds that exposed the panniculus carnosus and caused ulceration and inflammation. Daily local application of 0.1% TPL-ofi treatment, with normal saline (0.9% sodium chloride) as the control group. The results showed that 0.1% TPL-ofi significantly reduced wound edema, and granulation tissue was observed under the wound after 7 days of treatment. In addition, the occurrence of inflammation is often accompanied by the production of pain, and the mechanical threshold of animals treated with TPL-ofi increased significantly (123.6 ± 6.24 g vs saline = 106 ± 4.49 g). This suggests that TPL-ofi can reduce the clinical signs of inflammation. In the analysis of histopathological, it was found that the number of fibroblasts/myofibroblasts (2.4×) and blood vessels (1.9×) in the TPL-ofi group increased significantly with the increase of treatment time (37.8 ± 9.6 and 25.25 ± 4, respectively), while the degree of inflammatory infiltration gradually decreased. Myeloperoxidase (MPO) and glutathione (GSH) are inflammatory redox markers with proinflammatory and oxidative stress-reducing effects, respectively. Moreover, fibroblast growth factor (FGF)-2 has been shown to regulate many cellular functions including cell proliferation, migration, and differentiation, as well as angiogenesis in a variety of tissues, including skin, blood vessel, tendon/ligament, etc. Immunohistochemistry showed that TPL-ofi could increase the expression of FGF-2 immunoexpression (Adjafre et al., 2024). In summary, cactus polysaccharides play an important role in analgesia and anti-inflammation in skin wound models. It also provides a reference for the further development of safe and effective topical drugs.

### 4.3 Immunoregulatory activity

**FIGURE 4:**
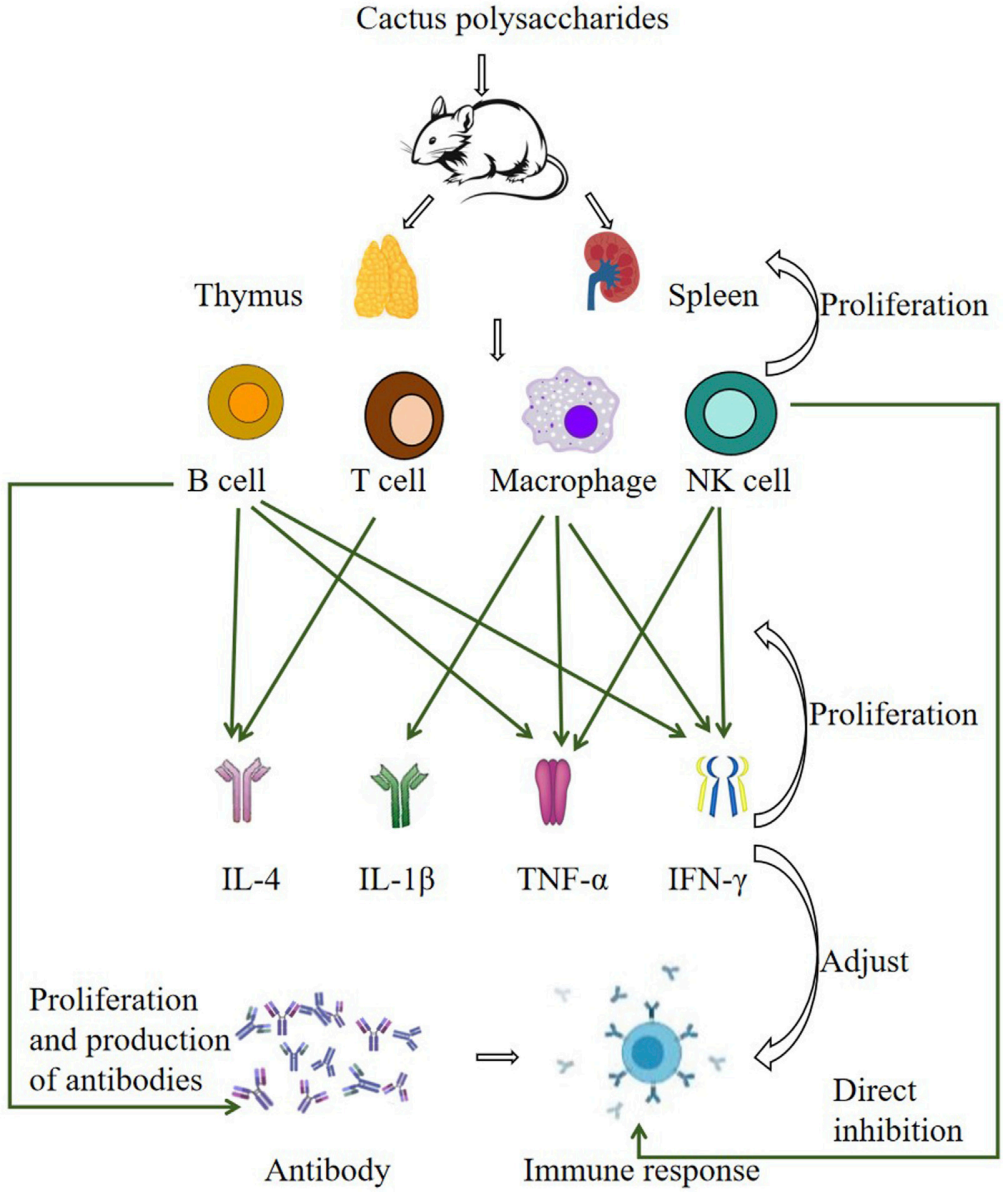
Mechanism of the immunomodulatory effect of cactus polysaccharides.

Polysaccharides can affect the function of the immune system through a variety of mechanisms, including the activity of immune cells, the production of cytokines (CK) and the regulation of immune responses. In recent years, plant polysaccharides have gradually attracted the attention of researchers due to their advantages of wide source and small side effects (Han et al., 2024). A polysaccharide ODPs was extracted from cactus and its regulatory effect on immunodeficient mice was evaluated. Immunodeficiency was induced by intraperitoneal injection of cyclophosphamide in mice. Three different doses of ODPs (100, 200 and 400 mg/kg) and the positive drug astragalus polysaccharides (APS) were used for treatment, while the blank group (BG) and the model group (MG) were given the same amount of normal saline. Blood analysis showed that ODPs could increase white blood cell counts in immunosuppressed mice. Spleen and thymus play an important role in the human immune system, and their indexes reflect the immune ability of the body to a certain extent. The results showed that compared with the MG, the ODPs treatment group could significantly increase the spleen and thymus index of the immunosuppressed mice, and the thymus index was close to the positive control (PC) group. CK is a small polypeptide or glycoprotein synthesized and secreted by a variety of tissue cells (mainly immune cells). It plays an important role in the regulation of cell-cell interaction, cell growth and differentiation in the process of immune response. In addition, studies have shown that polysaccharides can exert immunomodulatory activity by regulating the expression of CK. The levels of Interleukin-4 (IL-4), Interleukin-1*β* (IL-1*β*), tumor necrosis factor *α* (TNF-*α*) and interferon γ (IFN-γ) in plasma were measured by ELISA kit, and the effects of ODPs on immunosuppressive mice were analyzed. The results showed that medium and high doses of ODPs could significantly upregulate the levels of IL-4, IL-1*β*, TNF-*α* and IFN-γ, indicating that ODPs could improve the immune function of mice. Macrophage is an important immune cell, which plays an important role in the immune regulation of the body. The results showed that the clearance index and phagocytic index of the MG were significantly lower than those of the NG, indicating that the MG had immunosuppression. ODPs increased the clearance index and phagocytic index in a dose-dependent manner, thereby increasing the phagocytosis of mouse macrophages, and the therapeutic effect of the high-dose ODPs treatment group was comparable to that of the PC group. In addition, the effects of ODPs on some important metabolites, such as sinapyl alcohol, cafetol, adrenic acid, neocnidilide, dodecanedioic, maltol, *etc.*, were also studied. The results showed that cactus polysaccharides could affect the biosynthetic metabolic pathway of lysine. In summary, cactus polysaccharides can exert immunomodulatory activity by affecting the spleen and thymus index, affecting the expression of cytokines, phagocytosis of macrophages, and regulating metabolism (Liu et al., 2022) (Figure 4). These results provide a reference for further study of cactus polysaccharides, and at the same time provide important theoretical and practical basis for the development of new immunomodulators.

### 4.4 Other activities

The health benefits of cactus polysaccharides are diverse. In addition to the aforementioned biological activities, cactus polysaccharides also possess anti-glycation and anti-oxidant effects. Glycation reaction is one of the factors of aging and dark yellow skin. With the increase of age, along with the gradual slowing of metabolism, the amount of glucose intake in the body continues to accumulate, and finally forms advanced glycation end-products (AGEs). AGEs can accelerate the aging of the human body and cause the occurrence of many chronic degenerative diseases (Liu H. et al., 2024; Siddiqui et al., 2024; Irmak Aslan et al., 2024). Studies have shown that three polysaccharides (PCA, PCN and PCB) isolated from cactus by UAE have anti-glycation effect. Galactose-induced bovine serum protein was used as an experimental model to evaluate the anti-glycation activities of PCA, PCN and PCB, respectively, and aminosalicylic acid (ASA) was used as a PC. The results showed that when the concentration was 1 mg/mL, the inhibition rates of ASA, PCA, PCN and PCB were 82.7%, 76.7%, 69.9% and 55.9%, respectively. In conclusion, all three polysaccharides had anti-glycation effect *in vitro*, with PCA showing the strongest but lower inhibition than ASA (Chaouch et al., 2016). Oxidative stress is a negative effect produced by free radicals in the body, which is considered to be one of the important factors leading to a variety of diseases. Reactive oxygen species (ROS) is an important type of free radical, which can maintain the balance of cell metabolism and biochemical reactions under normal physiological conditions but can cause oxidative stress when excessive accumulation (Koletti et al., 2024; Li et al., 2024). PC, a pectin polysaccharide, was isolated from cactus cladodes and its anti-oxidant ability was confirmed by oxygen radical scavenging experiments. The results of *in vitro* experiments showed that PC could scavenge DPPH and ABTS radicals with a scavenging ability of 33.87% and 68.08%, respectively. In addition, *β*-carotene was associated with oxidative stress. The inhibition rate of *β*-carotene by PC was 69.91% (Bayar et al., 2016). Other studies have shown that polysaccharides UAEPC and EAEPC extracted from cactus cladodes have scavenging effects on DPPH radicals, while TPF obtained from this plant has good scavenging ability on hydroxyl radical (Lefsih et al., 2016; Bayar et al., 2017; Bayar et al., 2018). Several studies have shown that cactus polysaccharides also have anti-oxidant effect through a variety of oxygen free radical scavenging experiments (Chaouch et al., 2016; Salehi et al., 2019). To sum up, cactus polysaccharides have a potential as a natural anti-oxidant and have a broad application prospect.

## 5 Structure-activity relationships and structural modifications

A large number of studies have shown that the structure of polysaccharides is closely related to their biological activities, but the rules between the two are still not summarized. Continuing to explore the structure-activity relationship of polysaccharides is of great significance for the industrial application of polysaccharides (Chen et al., 2024; Fu et al., 2024). The structure of polysaccharides includes primary structure and advanced structure. The primary structure includes Mw, monosaccharide composition, sugar chain connection mode, sugar chain configuration, branching structure and so on. Advanced structures include molecular size, spatial conformation, condensed state characteristics and solution behavior. The structure of polysaccharides is closely related to the source of raw materials and the extraction and purification techniques. The Mw was closely related to the activity of the polysaccharides. TPL-ofi, a polysaccharide from cactus cladodes with a molecular weight of 227.781 kDa, has anti-inflammatory effect (Adjafre et al., 2024). In general, the larger the Mw of polysaccharides and the more active sites, the stronger the activity may be. However, too high molecular mass will affect the penetration of polysaccharides into the cell membrane, the cell interior and the target of action, and limit the biological activity of polysaccharides. Therefore, only the Mw distribution in a reasonable range is conducive to the biological activity of polysaccharides. PCA, PCN and PCB from cactus had different Mw of 9,220 ± 193.6, 7,770 ± 163.2 and 7,500 ± 127.5 kDa, respectively. Studies have shown that all three polysaccharides play an anti-oxidant role, among which PCA has the strongest activity (Chaouch et al., 2016). However, another polysaccharide PC obtained from cactus has three main Mw of 110.70 ± 3.37, 78.40 ± 5.49, 58.57 ± 4.31 kDa, which also exhibit activity in inhibiting oxidative stress (Bayar et al., 2016). Monosaccharide composition is a prerequisite for the study of polysaccharides, and also an important part of further exploring the structure-activity relationship. NwPS and NPec from cactus leaves were both composed of Ara, Gal, GalA, Glc, GlcA, Rha and Xyl in the ratios of 15 : 20: 27 : 18: 2 : 11 and 9 : 14: 44 : 15: 8 : 1: 9, respectively. Experiments confirmed that NwPS and NPec have the effect of promoting wound healing, but the effect on human keratinocytes and fibroblasts is different. This may be related to the different structure caused by the difference in glucose and glucuronic acid content between NwPS and NPec (Deters et al., 2012). In addition, the three polysaccharides PCA, PCN, and PCB in cactus all have antioxidant effects, with PCA having the strongest activity. Experiments have shown that the galacturonic acid rate of PCA is higher than that of PCN and PCB, which can more effectively resist free radicals and linoleic acid peroxidation (Chaouch et al., 2016). From this, it can be seen that different cactus polysaccharides contain different monosaccharide compositions, which can partially explain the differences in biological activity between different components of polysaccharides. However, it should be noted that the structure of polysaccharides is extremely complex, and these structures also have a certain impact on monosaccharide activity. Interpreting the overall biological activity of cactus polysaccharides solely based on monosaccharide composition has significant limitations, and its specific correlation mechanism still needs further exploration.

Mw can affect the physical and chemical properties of cactus polysaccharides, such as solubility and viscosity, thereby affecting their absorption in the body. Polysaccharides with high Mw have an inherent ability to enhance food viscosity. The Mw of OFIFG is as high as 3.67 × 10^6^ g/mol, with high viscosity and stability. Rheological analysis experiments have also confirmed this result, and it is expected to be added as a good thickener and stabilizer in food (Salehi et al., 2019). However, the high Mw and high viscosity of polysaccharides are also limiting reasons for their application. Compared to the high Mw cactus polysaccharide (6.8 × 10^6^ g/mol), the degraded cactus polysaccharide (1.4 × 10^4^ g/mol) has stronger antioxidant activity (Chaouch et al., 2016). Therefore, the size of Mw is closely related to the various biological activities of cactus polysaccharides. Further research should clarify the optimal Mw range for these biological activities to optimize the role of cactus polysaccharides in applications.

Due to the complexity of the polysaccharide structure, the current research on the structure of cactus polysaccharide is mainly on the primary structure. It is certain that the advanced structure plays a more important role in the activity of the polysaccharide. The structural analysis showed that there was rhamnose-galactoic acid I domain in EAEPC structure, which had antioxidant effect (Bayar et al., 2018). In addition, NMR analysis showed that the cactus polysaccharide CSP3 is composed of repeating units →2)-*α*-l-Rha*p*-(1→4)-*α*-d-Gal*p*A-(1→, with 71% of rhamnose units linked in O-2 and 29% both in O-2 and O-4, and with side chains of either arabinan (average of 25–30 arabinose units) or galacto-oligosaccharides (average of 3–4 galactose units) attached to O-4 of the backbone rhamnose units, but its health benefits have not been studied (Habibi et al., 2004a). In the future, the structural characteristics and biological activities of cactus polysaccharides should be further explored to lay a foundation for better elucidating the relationship between the two and their product development and industrial development.

In order to better exert the biological activity of polysaccharides, researchers modify the structure of polysaccharides appropriately. At present, chemical modification, physical modification and biological modification are the main methods of structure modification. Among them, chemical modification is the most commonly used method, including sulfation, carboxymethylation, acetylation and phosphorylation. The researchers used SO_3_-DMF complex to sulfate cactus polysaccharide (PC) to obtain two sulfated polysaccharides (PCS_1_, PCS_2_), of which PCS_2_ had the highest sulfate content of 7.04%. It has been reported that sulfate groups are strongly associated with anticoagulant capacity. PCS_1_ and PCS_2_ showed potent anticoagulant activity after prolonged activated partial thromboplastin time (APTT) and thrombin time (TT), while PC without sulfation had no anticoagulant effect. This indicates that the introduction of sulfate group in the polysaccharide seems to be the main factor for anticoagulant activity (Chaouch et al., 2018). At present, there are few studies on the structural modification of cactus polysaccharide. In the future, we will focus on the structural modification of cactus polysaccharide and the effect of modification on biological activity, so as to provide a basis for the discovery of new drugs.

## 6 Application of cactus polysaccharides

**FIGURE 5:**
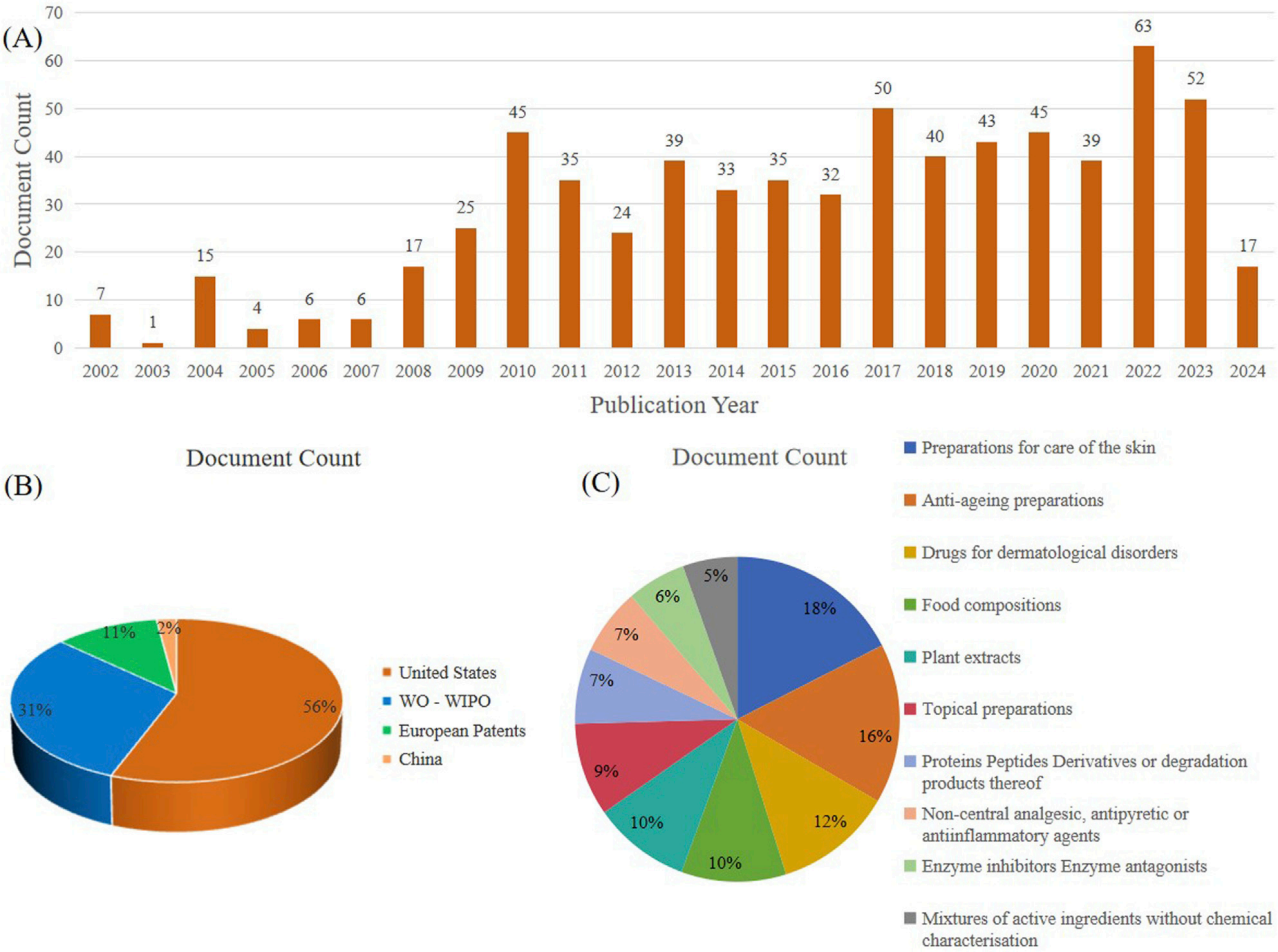
Analysis of cactus polysaccharides patents searched in www.lens.org: **(A)** Number of patent applications per year. **(B)** Jurisdiction **(C)** Central product classification.

Polysaccharides are large molecular polymers that exist in nature and have the advantages of high stability and good biocompatibility. They have always been a hot topic in the application research of the food industry, biomedical industry, and daily cosmetics industry (Liao et al., 2023; Yang et al., 2024). With the increasing research on cactus polysaccharides, their activity studies are also becoming more and more abundant. As a result, there are increasingly more patents related to cactus polysaccharides. At present, there are 673 patents on cactus polysaccharides in the world. Figure 5 provides a detailed analysis of the current status of patented inventions related to cactus polysaccharides in recent years. The analysis showed that the US and the World Intellectual Property Organization (who-wipo) accounted for the majority of patents, accounting for 56% and 31%, respectively, while Europe and China accounted for a smaller share of 11% and 2%, respectively.

The data shows that cactus polysaccharides have broad application prospects in the pharmaceutical industry. A composition containing cactus polysaccharides was invented for the treatment of hemorrhoids and related diseases. Modern pharmacological studies show that cactus polysaccharides have a good anti-inflammatory effect, which also explains its therapeutic effect on hemorrhoids. Gastroesophageal Reflux Disease (GERD), which is the mucosal injury and chronic syndrome caused by abnormal reflux to the esophagus, is clinically characterized by heartburn, sour regurgitation, swallowing pain and swallowing difficulties, which seriously affect people’s healthy life. A combination containing *Opuntia Ficus Indica* cladodes polysaccharides and other extracts, which can be used for the prevention and treatment of GERD and the upper gastrointestinal tract diseases. In addition, the green and nutritious cactus lozenges developed with edible and medicinal plants as raw materials contain rich plant polysaccharides, which can enhance and improve the nutritional diet and healthcare effect of patients with type 2 diabetes and help to improve the healthy living standard of patients with diabetes. Cactus lozenges are mainly composed of 40–50 parts of cactus, 15–20 parts of jerusalem artichoke, 10–15 parts of chicory, 8–12 parts of kudzu-vine roots, 5–10 parts of *Stevia rebaudiana* and 2-3 parts of liquorice roots. The synergistic reactions of the cactus and the Chinese herbal medicines are utilized to effectively improve the nourishing and health-caring functions of the lozenges, and cactus lozenges are safe, free of side effects and convenient to eat, and have relatively good market application prospects.

Cactus polysaccharide has good water retention and anti-oxidant ability, and it has made significant progress in the daily chemical industry. Researchers have created a cosmetic that can improve the function of the skin. First, *Ecklonia cava* extract treated with the low Mw of polysaccharide to a monosaccharide and oligosaccharide by inoculating yeast after sterilizing *E. cava* at high temperature and pressure, and then the cactus extract by electrolyzing cactus mucilage which is obtained in a cactus raw material (root, stem, and fruit) in an electrolyte solution. Finally, *E. cava* extract and cactus extract are mixed with manihot starch to make a cosmetic composition for skin moisturizing. cactus extract can improve skin function and relieve atopic dermatitis. In addition to the skin, the lips are also a place worth protecting. Lip balm is a commonly used product for lip care in daily life. It can play a good role in relieving chapped lips. The lip balm made of cactus as the main raw material was prepared. The advantage of this lip balm is that the polysaccharides contained in cactus can achieve moisture preservation effects, metabolism of the lips of the users can be promoted if the lip balm is used for a long time, melanin can be prevented from being precipitated, anti-aging and anti-wrinkle effects and the like can be realized, and moisture can be locked in deep layers. Furthermore, the cactus contains abundant vitamins A and B and diversified small-molecule amino acid and is extremely high in oxidation resistance, cell regeneration can be promoted, the softness of the lips of users can be improved. In conclusion, cactus polysaccharide has a broad development prospect in the pharmaceutical and cosmetic industries, and health products containing cactus polysaccharide should be further developed in the future.

## 7 Conclusion and future perspectives

Cactus is a natural plant that can be both edible and medicinal. Polysaccharides can be extracted from multiple parts of cacti, including cladodes, leaves, fruits, fruit peel, and pericarp seeds. The different extraction parts result in different structures of cactus polysaccharides obtained. d-xylans were extracted and isolated from pericarp seeds, which can be used as a dietary fiber supplement, a sustained-release drug delivery system in the pharmaceutical industry, and a stabilizer and thickener in the food industry. Polysaccharides from cladodes are mainly pectin polysaccharides, which have numerous applications in various fields such as food and pharmaceuticals. The structure and activity are closely related, and the various pharmacological effects of these polysaccharides have been proven. They can promote wound healing, as well as have anti-inflammatory, immunomodulatory, antioxidant, and anti-glycation effects. It can be seen that polysaccharides may be a good source for future pharmacological research and the development of new supplements. The new strategic research on developing value-added products with the potential of cactus polysaccharides may be particularly interesting, which also opens up new prospects for developing products with health benefits.

Despite some progress in the research of cactus polysaccharides, there are still many opportunities and challenges that need to be addressed urgently. Firstly, existing extraction methods have shortcomings in extracting cactus polysaccharides. In the future, new extraction technologies such as ultra-high pressure, ultrasound, supercritical fluid extraction, and subcritical water extraction or a combination of multiple technologies can be used to extract cactus polysaccharides, achieving environmentally friendly, high-yield, and low energy consumption. We should compare the effects of different extraction methods on the yield, structural characterization, and biological activity of cactus polysaccharides to establish standardized extraction and separation methods (Wang et al., 2022; Wei et al., 2023; Li et al., 2023). Standardized method specifications will help ensure consistency of cactus polysaccharides from different sources. In addition, the scalability of the extraction process for potential industrial applications should also be considered. This is also an issue that needs to be considered for purification. The most important thing is to maintain the biological activity of polysaccharides during the extraction and purification process. Because the activity of polysaccharides is closely related to their structure, high temperatures or other extreme conditions during the extraction process may damage the structure of polysaccharides, thereby affecting their activity. Secondly, the structural analysis of cactus polysaccharides requires more advanced analytical tools to elucidate, such as X-ray diffraction and atomic force microscopy (Lin et al., 2024; Liu J. et al., 2024; Chen et al., 2024). The relevant reports on X-ray diffraction and atomic force microscopy have not yet involved the study of cactus polysaccharides, but the application of these technologies will help to further elucidate their structure in the future.

Cactus polysaccharides have a wide range of biological activities. However, most studies have only reported their *in vitro* biological activities, and their mechanisms of action lack in-depth research. In addition, the lack of pharmacokinetic studies on cactus polysaccharides also limits their application. Therefore, further research, including clinical studies, should have a deeper understanding of the mechanism of cactus polysaccharides. In addition, modern pharmacological research has shown that cactus polysaccharides have the effect of promoting wound healing. Based on this effect, cactus polysaccharide and chitosan/polyvinyl alcohol (PVA) can be used to prepare hydrogel for wound healing. The hydrogel designed with cactus polysaccharide has excellent physical and chemical properties and unique therapeutic intervention, which can effectively protect the wound from bacterial invasion while promoting wound healing. More research is needed to design cactus polysaccharide hydrogels in the future to realize the great potential of wound healing.

Functional foods are gradually being favored by people, especially health foods mainly composed of cactus polysaccharides, which have broad market prospects and commercial value in enhancing immunity and antioxidant properties. They can be further explored and explored in this field, making contributions to the development of food and health products. Currently consumers not only demand safe, healthy, and stable food, but also demand natural, sustainable, high-quality, and safer food packaging. Based on this, people’s interest and research activities in edible packaging continue to increase. Cactus polysaccharides have the characteristic of forming edible films and have great potential for development (Gheribi et al., 2019). However, the research and production of edible films are currently immature and rarely put into the market, which has not yet met people’s needs and requires further research. In the field of medicine, there have been many applications of polysaccharides, but the application of cactus polysaccharides is not yet widespread and mainly focuses on antioxidant and anti-inflammatory aspects. With the continuous deepening of research work and the increasing demand for natural source substances, the deep processing technology of cactus polysaccharides will continue to improve, and its product development will become a key research topic in the future.

This article reviews the research progress of cactus polysaccharides in recent years and points out the important value of cactus polysaccharides in the fields of medicine and cosmetics. Although this article has made efforts to summarize existing research, there are still some limitations. Firstly, this review may not cover all relevant studies, such as those that have not been extensively indexed and the latest research findings. Secondly, the data sources of some studies are not extensive enough, which may lead to limitations in the results, especially in pharmacological experiments. Despite the limitations mentioned above, this review can also provide valuable references for future research directions. With more comprehensive and in-depth research on cactus polysaccharides, it is believed that cactus polysaccharides have broad application prospects and markets.
